# Editorial: The role of pericytes in physiology and pathophysiology

**DOI:** 10.3389/fphys.2023.1306031

**Published:** 2023-10-20

**Authors:** Shaun L. Sandow, Sean M. Wilson, M. Dennis Leo

**Affiliations:** ^1^ Biomedical Science, School of Health, University of the Sunshine Coast, Maroochydore, and Center for Clinical Research, Faculty of Medicine, University of Queensland, Brisbane, QLD, Australia; ^2^ Division of Pharmacology, School of Medicine, Loma Linda University, Loma Linda, CA, United States; ^3^ Department of Physiology, University of Tennessee Health Science Center, Memphis, TN, United States

**Keywords:** endothelium, capillary, blood flow, smooth muscle, exchange, pericyte

**FIGURE 1:**
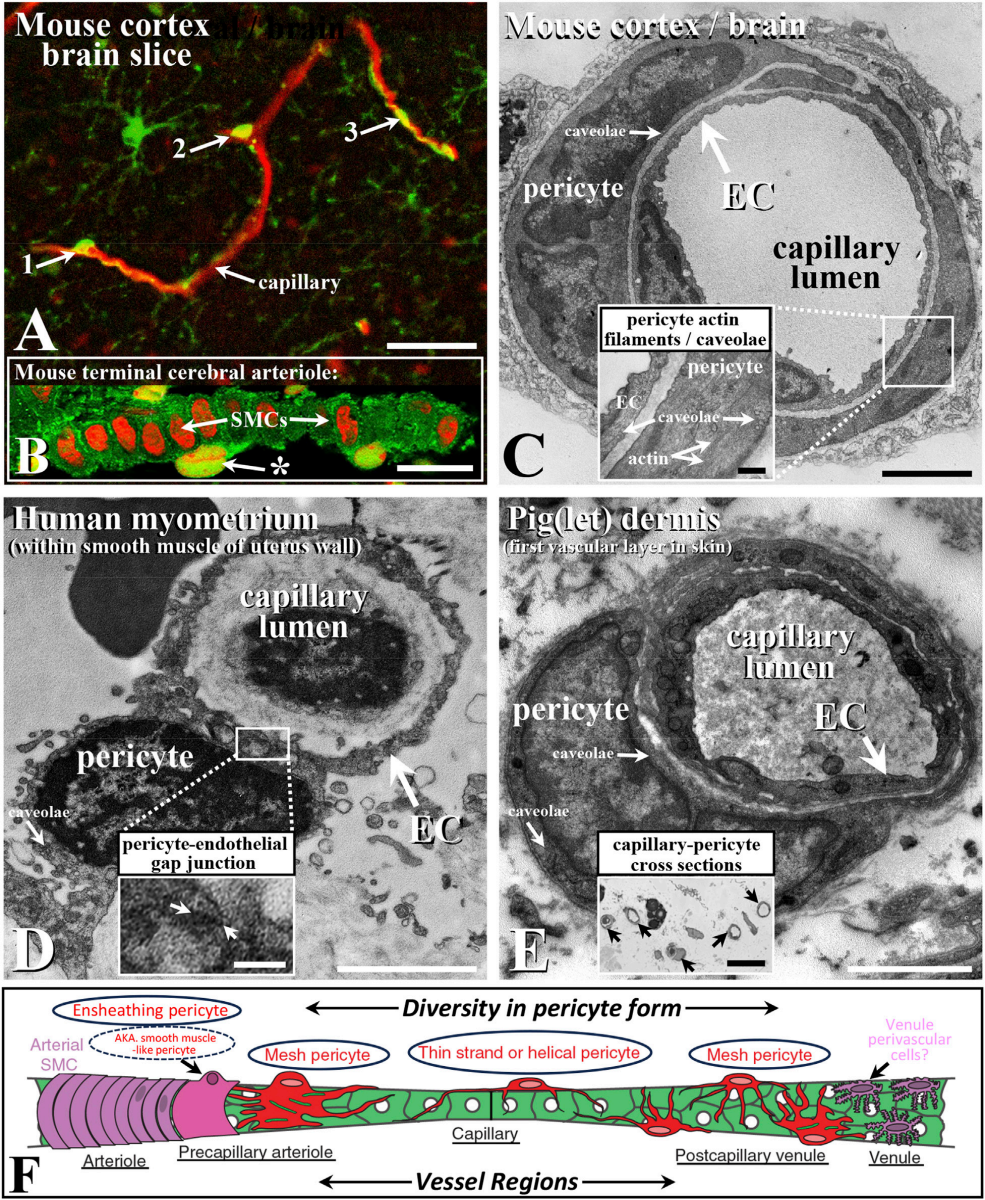
Confocal immunohistochemical **(A,B)** and ultrastructural **(C–E)** characteristics of capillaries and their pericytes **(A,C–E)** and terminal arteriole pericyte-like cells **(B,F)** with schematic **(F)** demonstrating diversity in species capillary bed pericyte structure (e.g., Hartmann et al., 2021; Abdelazim et al., Hartmann et al., 2022; Davis and Attwell, 2023; Preishuber-Pflügl et al.). Capillaries (red; blood vessel/microglia-targeted lectin) in mouse cortex brain slice with examples of morphologically distinct pericytes **(A)**; examples 1-3, arrows; green, platelet-derived growth factor; modified from/see Staehr et al., 2023, for label detail). Mouse cerebral arteriole **(B)**; caveolin-1, green; propidium iodide, red; from Hinkley et al., 2023b) with pericyte-like cell (*) external to the smooth muscle cells (SMCs), with their long axis perpendicular to vessel length axis (left to right) and capillary from mouse cortex **(C)**; Hinkley et al., 2023a; see also Bonney et al., 2022), noting actin filaments typically associated with contractile SMCs (Sandow et al., 2006; Figure 5) as a likely ensheathing pericyte **(C)**, inset; Hartmann et al., 2022) and high densities of caveolae (inset; see comparative Howitt et al., 2012) as sites of caveolin-1 scaffolding protein and as key microdomain signaling sites (Grayson et al., 2013; Kenworthy et al., 2023). Human myometrial/uterine capillary **(D)**; from Luque et al., 2022) with heterocellular gap junctions (boxed/arrows; **(D)**, inset), typical in some pericyte-EC associations, and pig epidermis/skin capillary and pericyte **(E)**; from Hinkley et al., 2023a, noting numerous dermal capillary cross sections; **(E)**, inset). Endothelial cells (ECs) line the vessel lumen, with adjacent external pericytes **(C–E)**. Schematic of microvascular pericyte structural diversity typical of many vascular beds (as per above, e.g., citations), associated with arteriole > capillary > venule progression in mouse cerebral microvasculature **(F)**; circled examples; modified from Hartmann et al., 2015; Hartmann et al., 2022). Bar, **(A)**, 25 μm; **(B,E)** inset 10 μm; **(C–E)**, 2 μm; **(C)**, inset, 0.5 μm; **(D)**, inset, 0.25 μm. Images modified/reproduced from the above citations with permission.

In this Research Topic of *Frontiers in Physiology*, studies outline some of the diverse structural and functional properties of capillary and non-capillary pericytes and pericyte-like cells in health and disease. Capillaries are the site of gas, nutrient, and waste exchange between the blood, cells, and tissues and are located between the arteriole–venule blood supply. The primary role of pericytes is to wrap around regions of the tubular capillary endothelium at intermittent sites, where they contribute to matching metabolic demand to blood flow requirements. This role of pericytes/pericyte-like cells is accompanied by matched structural specialization and diversity in different vascular beds, tissues, and species, as well in development and ageing, as per specific physiological and pathophysiological conditions. In addition to their characterized direct vascular role (Abdelazim et al., Nakisli et al.; Figure 1), including in capillary formation (Kemp et al.), such cells confer non-exclusive and potentially exclusive, selective, and specific functions, encompassing novel roles such as modulating immune cell function, glucose homeostasis, and wound healing (Burganova et al., Preishuber-Pflügl et al.). Notably, a specific molecular marker for pericytes remains elusive (Abdelazim et al., Preishuber-Pflügl et al.).

Three broad types of morphologically distinct pericytes have been identified and are best characterized in the brain vasculature as mesh, thin strand-helical, and ensheathing (Attwell et al., 2016; Beard et al., 2020; Hartmann et al., 2015; Hartmann et al. 2021; Hartmann et al. 2022; Figure 1). As per their name, thin- (single or paired) strand-helical pericytes exhibit twisting processes that can extend along regions of the capillary network. These processes potentially contact and perhaps form electrical and metabolic coupling sites via heterocellular gap junctions with multiple endothelial cells. Mesh pericytes are suggested to predominate at the venule and arteriole–capillary interface regions (Figure 1), while ensheathing pericytes (as smooth muscle (SM)-like pericytes) share aspects of a SM/contractile phenotype (containing SM-α actin and myosin) and are located near the arteriole–capillary interface (Figure 1), with structural–functional specialization as a primary property.

Based on distinct ultrastructural characteristics and consistent with the above, with a focus on the cerebral vasculature, Abdelazim et al. classify three primary types of pericytes as thin-strand, mesh, and ensheathing (see also Figure 1; Attwell et al., 2016; Gonzales et al., 2020; Bonney et al., 2022; Hartmann et al., 2015; Hartmann et al. 2021; Hartmann et al. 2022). Notably, they also describe an apparently distinct ensheathing type of cell between endothelial and SM cells at the capillary–arteriolar interface. These cells are again consistent with previous reports of a pericyte-like contractile cell that is involved in shunting blood flow in at least some and perhaps many/all vascular beds, including in cerebral capillaries (e.g., above citations; Staehr et al., 2023; Figure 1), containing what is presumably SMα actin as part of the contractile apparatus; hence, some of the debate on the naming of this cell, with ensheathing or “pericyte-like” being a reasonable hedge and reflecting the ongoing debate on pericyte nomenclature. The authors also note the potential for direct pericyte–endothelial contact, which has been previously described, with various roles attributed to this heterocellular coupling (Figure 1). These roles include coordinating electrical coupling and the local transfer of second messengers, such as inositol-1,3,5-trisphosphate and cAMP. Indeed, such endothelial–pericyte contacts include the ‘peg and socket’ form described by Abdelazim et al. between the cerebrovascular and parenchymal cells, perhaps as specific communication sites and/or for mechanical connectivity.

Nakisli et al. review mural/pericyte-like cell diversity in arteriovenous malformations (AVMs) of the CNS with a focus on pathology-related changes therein. Mural cell pericytes, which exhibit similarity and analogous features to the *pericyte-like* contractile cell described by Abdelazim et al. and many others (see previous citations and Figure 1), are described in AVMs, where the AV connection is widened, facilitating increased blood flow and pressure (BP). Nakisli et al. clarify the alterations in such cells and associated AVMs with underlying neurovascular dysfunction in several disease states. Consistent with the literature, they note that there is significant heterogeneity in the molecular, cellular, and functional characteristics of these cells, which likely reflect specific function and dysfunction in health and disease (e.g., Brown et al., 2019; Nakisli et al.; Preishuber-Pflügl et al.). They also clarify AVM formation in many tissues, with elevated BP leading to microhemorrhages, inadequate nutrient–waste exchange, and vessel tortuosity and entanglement as a “nidus” (Latin for nest). Of significant value are the literature summary (Tables 1, 2) shown in this study that provide insight into the changes to pericyte/pericyte-like and SM cells associated with CNS AVMs.

Preishuber-Pflügl et al. clarify the role of pericytes in wound healing and scar formation with a focus on brain and spinal cord injury, including the use of a murine optic nerve crush (ONC) lesion model. As broadly acknowledged, the lack of a selective pericyte identifier makes comparative study problematic (citations above), with the contractile pericytes or “pericyte-like” cells being an appropriate example. They focus on use of an inducible PDGFRβ-P2A-CreERT2-td tomato lineage tracing reporter to demonstrate the role and fate of pericyte-derived cells in ONC injury. Non-vascular associated pericyte-derived cells are found to be associated with fibrotic scar tissue formation post-ONC injury. Like many pericyte-related activities, the authors suggest that such cells may be a target for therapy; in this case, to improve axonal regeneration and correct fibrotic scar formation.

Burganova et al. show that pericytes associated with pancreatic beta-islet cells produce the cytokine interleukin-33 (IL-33), thus being essential for glucose homeostasis. In an IL-33 selective pericyte gene deletion transgenic mouse model, the authors have characterized pancreatic production of this cytokine by measuring the *in vivo* and *in vitro* response to glucose and recombinant IL-33 administration, as well as beta-cell mass and gene expression. Interestingly, the potential critical importance of pancreatic pericytes was implied whereby mice lacking pericyte IL-33 were glucose intolerant through impaired insulin secretion, with reduced beta islet T- and dendritic cell numbers and lower retinoic acid production by islet macrophages. Thus, they suggest that pericytes are an integral part of islet architecture, regulating immune cell activity to support IL-33-dependent beta-cell function.

As an essential process for vascular health and disease, Kemp et al. examine pericyte function in capillary tube network assembly. Understanding the signaling pathways that control endothelial tube formation and the related development of the extracellular matrices, including the expression of specific matrix proteins, cytokines, hormones, and growth/angiogenic factors, is a topical research area. The appearance of pericytes on the abluminal surface of endothelial tubes can result in narrowing and elongation of the capillary lumen, whereas their absence results in a lack of these processes and exacerbation of tube diameter and reduced capillary length. Notably, endothelial presence and their production of various growth factors including ET1 are required for pericyte recruitment and action in tube development, perhaps through regulation of the matrix (Meehan et al., 2016).

The studies outlined here and published in this Research Topic of *Frontiers in Physiology* detail some of the diversity in structure, function, and location properties of pericyte and pericyte-like cells. Much future work is required on this widely distributed cell with diverse phenotypes, although it is clear that there is significant heterogeneity in pericyte form and function; thus, it is essential to further define their specific features in health and disease.
